# Mechanism and Prevention of Titanium Particle-Induced Inflammation and Osteolysis

**DOI:** 10.3389/fimmu.2018.02963

**Published:** 2018-12-18

**Authors:** Michal Eger, Sahar Hiram-Bab, Tamar Liron, Nir Sterer, Yaron Carmi, David Kohavi, Yankel Gabet

**Affiliations:** ^1^Department of Anatomy and Anthropology, Sackler Faculty of Medicine, Tel Aviv University, Tel Aviv, Israel; ^2^Department of Prosthodontics, Goldschleger School of Dental Medicine, Sackler Faculty of Medicine, Tel Aviv University, Tel Aviv, Israel; ^3^Department of Pathology, Sackler Faculty of Medicine, Tel Aviv University, Tel Aviv, Israel

**Keywords:** dental implants, peri-implantitis, macrophages, cytokines, osteoclasts

## Abstract

The worldwide number of dental implants and orthopedic prostheses is steadily increasing. Orthopedic implant loosening, in the absence of infection, is mostly attributable to the generation of wear debris. Dental peri-implantitis is characterized by a multifactorial etiology and is the main cause of implant failure. It consists of a peri-implant inflammatory lesion that often results in loss of supporting bone. Disease management includes cleaning the surrounding flora by hand instruments, ultrasonic tips, lasers, or chemical agents. We recently published a paper indicating that US scaling of titanium (Ti) implants releases particles that provoke an inflammatory response and osteolysis. Here we show that a strong inflammatory response occurs; however, very few of the titanium particles are phagocytosed by the macrophages. We then measured a dramatic Ti particle-induced stimulation of IL1β, IL6, and TNFα secretion by these macrophages using multiplex immunoassay. The particle-induced expression profile, examined by FACS, also indicated an M1 macrophage polarization. To assess how the secreted cytokines contributed to the paracrine exacerbation of the inflammatory response and to osteoclastogenesis, we treated macrophage/preosteoclast cultures with neutralizing antibodies against IL1β, IL6, or TNFα. We found that anti-TNFα antibodies attenuated the overall expression of both the inflammatory cytokines and osteoclastogenesis. On the other hand, anti-IL1β antibodies affected osteoclastogenesis but not the paracrine expression of inflammatory cytokines, whereas anti-IL6 antibodies did the opposite. We then tested these neutralizing antibodies *in vivo* using our mouse calvarial model of Ti particle-induced osteolysis and microCT analysis. Here, all neutralizing antibodies, administered by intraperitoneal injection, completely abrogated the particle-induced osteolysis. This suggests that blockage of paracrine inflammatory stimulation and osteoclastogenesis are similarly effective in preventing bone resorption induced by Ti particles. Blocking both the inflammation and osteoclastogenesis by anti-TNFα antibodies, incorporated locally into a slow-release membrane, also significantly prevented osteolysis. The osteolytic inflammatory response, fueled by ultrasonic scaling of Ti implants, results from an inflammatory positive feedback loop and osteoclastogenic stimulation. Our findings suggest that blocking IL1β, IL6, and/or TNFα systemically or locally around titanium implants is a promising therapeutic approach for the clinical management of peri-implant bone loss.

## Introduction

The prevalence of orthopedic and dental titanium (Ti) implants has increased steadily worldwide. Despite high success rates during the first 10 years (1, 2), orthopedic loosening and oral peri-implantitis remain a major problem for clinicians and constitute a major health problem for the profession. As mentioned, dental peri-implantitis is the main cause of implant failure. The risk of peri-prosthetic complications after 10 years ranges from 20 to 56% (3, 4); however, currently there are no acceptable, standardized protocols for treatment and consequently, recurrence rates remain high (5). Increasing the life expectancy of Ti prostheses is thus a major challenge in orthopedics and oral rehabilitation.

Oral peri-implantitis is believed to have a microbial etiology. However, a strong body of evidence links implant failure to aseptic inflammation around implants due to shedding of debris, Ti ions, and particles (1). Similarly, aseptic loosening of orthopedic implants has been attributed to Ti debris and particles (6). Although Ti prostheses are very biocompatible, these Ti byproducts are far from being bio-inert. Soft tissue biopsies around failing orthopedic (7, 8) and oral (9–11) implants revealed severe inflammatory reactions around aggregates of Ti particles.

We have previously shown that Ti particles released from ultrasonic (US) scaling around dental implants induce a marked inflammatory response in macrophages, with increased expression of pro-inflammatory cytokines, mainly IL1β, IL6, and TNFα. These Ti particles activate osteoclastogenesis *in vitro* and trigger inflammatory bone resorption *in vivo* (12).

Our previous results led us to further investigate the mechanism by which Ti particles entrain bone resorption and to investigate the therapeutic potential of neutralizing antibodies against IL1β, IL6, or TNFα in preventing Ti particle-induced osteolysis.

## Materials and Methods

All procedures involving animals were carried out in accordance with the guidelines of Tel Aviv University and were approved by the Institutional Animal Care and Use Committee (permit number M-015–047).

### Cell Culture

Primary bone marrow-derived macrophages (BMDMs) were isolated from the femora and tibiae of adult C57BL/6J mice (Envigo, Israel), as previously described (13). Briefly, cells were cultured overnight in 6-well dishes at 37°C in a humidified atmosphere with 5% CO_2_ in our “standard medium” consisting of alpha-modified Eagle's medium (αMEM, Life Science Technology, NY, USA) and 10% fetal bovine serum (FBS, Rhenium, Ltd, Modi'in, Israel). After 24 h, the non-adherent fraction was cultured in 10-cm non-culture-treated dishes containing standard medium and 100 ng/ml macrophage colony stimulating factor (M-CSF), prepared as previously described (14). The resulting adherent BMDMs were collected after 3 days for the specific assays described below.

### Particle Generation

To obtain Ti particles that correspond to the particles shedding from oral implants during routine scaling, we subjected Ti discs that were made from Ti6Al4V (AlphaBio Tec., Petah-Tikva, Israel) to ultrasonic scaling (Newtron Led, Satelec, Acteon, Marignac, France), adjusted to a frequency of 32 kHz. Particles were obtained from discs with a machined (M), sand-blasted and acid-etched (SLA) or sand-blasted (SB) surface topography as described previously (12). When not specified, SLA-derived particles were used. All particles were generated in a sterile environment. Each disc was subjected to US scaling for 60 s in distilled water (ddH2O), then cleaned twice with ethanol, and finally resuspended in distilled water. We previously showed that each 6 mm diameter disc generates ~2.54 million particles on average. In all our *in vitro* assays and for the preparation of the fibrinogen-thrombin membranes (see below) we used a particle density of 1,293 particles/mm^2^.

### Environmental Scanning Electron Microscopy (E-SEM)

To examine the cellular response of macrophages to Ti particles, BMDM were seeded on glass slides in a 10-cm plate (10^6^ cells per well) and cultured for 24 h in the presence of Ti particles released by the US scaling of SLA-treated discs. Cultures were then visualized by E-SEM. Each field was taken either in backscattered electrons mode (BSE) or secondary electrons (SE) mode to distinguish between extracellular vs. total Ti particles in the culture, respectively.

### Protein and Nitric Oxide (NO) Measurements in Conditioned Medium, RNA Isolation, and RT-qPCR

Following a 24–48 h incubation with Ti particles (or LPS or vehicle only), the supernatant was collected and referred to as conditioned medium (CM). Secreted protein amounts of pro- and anti-inflammatory cytokines in CM were measured using a multiplex assay and expressed in MFI units (Multiplex Fluorescent Immunoassay, ProcartaPlex Multiplex Immunoassay, eBioscience, San Diego, CA, USA). NO content was measured using the Griess Reagent System kit (Promega, Madison, WI, USA). After collecting the supernatant, macrophages were washed with sterile PBS, and RNA was extracted using Tri-RNA Reagent (Favorgen Biotech Corp, Kaohsiung, Taiwan). The 260/280 absorbance ratio was measured to verify the RNA purity and concentration. cDNA was produced using a high-capacity cDNA reverse transcription kit (Invitrogen, Grand Island, NY, USA), and real-time PCR was performed using Kapa SYBR Fast qPCR (Kapa Biosystems, Wilmington, MA, USA) on a StepOne real-time PCR machine (Applied Biosystems, Grand Island, NY, USA). We examined the expression of IL1β, IL6, and TNFα, which are established markers of macrophage inflammation. The primer sets were as follows: F-GAA ATG CCA CCT TTT GAC AGTG and R-TGG ATG CTC TCA TCA GGA CAG for mouse IL1β; F-TAG TCC TTC CTA CCC CAA TTT CC and R-TTG GTC CTT AGC CAC TCC TTC for mouse IL6; and F-TCT TCT CAT TCC TGC TTG TGG and R-GGT CTG GGC CAT AGA ACT GA for mouse TNFα. The reaction was subjected to 40 cycles of amplification using the following program: 95°C for 20 s, 60°C for 20 s, and 72°C for 25 s. The relative mRNA expression levels of the selected genes were normalized to the level of GPDH, which was amplified using the following primers: F-ACC CAG AAG ACT GTG GATG G and R-CAC ATT GGG GGTA GG AACAC.

### Osteoclastogenesis Assay

Preosteoclasts, prepared like the BMDMs, were plated in 96-well plates (7,000 cells per well) in standard medium supplemented with 20 ng/ml M-CSF and 50 ng/ml Receptor Activator of Nuclear Factor Kappa-B Ligand (RANKL) (R&D Systems, Minneapolis, MN, USA). On the 3rd day (after 48 h), the medium was replaced by the CM of BMDM, supplemented with RANKL and M-CSF. Where indicated, we also added 2 μg/ml of neutralizing antibodies (Ab) against IL1β (Kineret, anakinra, SOBI, Stockholm, Sweden), IL6-receptor (Actemra, tocilizumab, Roche, San Francisco, CA, USA), or TNFα (Humira, adalimumab, AbbVie Inc., Chicago, IL, USA). These neutralizing Ab, effective in both humans and mice (15–19), are referred to as anti-IL1β, IL6, and TNFα Ab, respectively. On the 4th day, cells were stained using a TRAP kit (Sigma-Aldrich, St. Louis, MO, USA), and multinucleated (>3 nuclei) TRAP-positive cells were defined as osteoclasts. Images were acquired at an original magnification of × 4 (Evos FLC, Life Technologies, MS, USA). The number of osteoclasts and the total osteoclast area were measured using ImageJ software (National Institutes of Health, Bethesda, MD, USA).

### Animal Model and Micro-Computed Tomography (μCT)

We used our calvarial model, which was described before (12). Briefly, US-released Ti particles (from M/SLA/SB-treated discs) were incorporated into a fibrinogen-thrombin degradable membrane used as a scaffold to localize the Ti particles. Membranes with no particles or only LPS were prepared as positive and negative controls. As indicated, neutralizing antibodies against TNFα (or saline as control) were incorporated into the membrane together with the Ti particles.

After anesthesia, the skin of 10-week-old C57Bl/6J female mice was shaved and disinfected. The parietal bones of the mice were exposed via a 10-mm incision in the nape area, and the periosteum was removed using a periosteal elevator. The fibrinogen-thrombin membranes were inserted to cover both parietal bones. The surgical incision was then closed using nylon monofilament surgical sutures (5/0). In the sham controls, no membranes were inserted and the incisions were closed. Another control group consisted of inserting an empty fibrinogen-thrombin membrane (with no Ti particles).

To test the therapeutic potential of systemic administration of neutralizing antibodies, we injected antibodies against IL1β, IL6, or TNFα (or saline as control) intraperitoneally 1 day before and once a week (2 mg/kg IL6 and 10 mg/kg TNFα) or daily (10 mg/kg IL1β) after membrane insertion, in accordance with the established clinical procedures (20–23).

All groups comprised a minimum of 6 animals. Animals were euthanized at the indicated post-operative time, and the skull of each mouse was removed, fixed for 24 h in 4% phosphate-buffered formalin, followed by 70% ethanol. All specimens were scanned and analyzed using a μCT system (μCT 50, Scanco Medical AG, Switzerland). Scans were performed at a 10-μm resolution in all three spatial dimensions, with 90 kV energy, 88 μA intensity, and 1,000 projections at a 1,000 m s integration time. The region of interest (ROI) was defined as two 3.7-mm circles in the center of the parietal bones. A custom-made algorithm, based on Image-Processing Language (IPL, Scanco Medical), was developed to isolate the resorption pits, defined as unmineralized pits that were 10 to 40-μm deep on the bone surface (12). Morphometric parameters were determined at the 3D level and included the total volume of bone pits (Pit Resorption Volume, PRV, mm^3^) and the bone tissue volume inside ROI (TV, mm^3^), which was used to determine the PRV/TV (%).

### FACS Analyses

Using our calvarial model, we also determined the effect of Ti particles on macrophage polarization, *in vivo*. The mice received either control membranes (fibrinogen-thrombin only) or membranes including LPS/Ti particles. Mice were euthanized 3 weeks post-op. This time point was chosen to be after the acute inflammatory response induced by the surgery but before the potential resolution of the inflammatory response to the Ti particles. The soft tissue covering the parietal bones was collected and processed using collagenase. Cells were labeled using specific markers (CD11b, Ly6-C, Ly6-G, F4/80, and MHC-II). This panel was used to define macrophages and to differentiate between M1 (CD11b+, Ly6C+, Ly6G+, F4/80+, MHC-II+) and M2 (CD11b+, Ly6C-, Ly6G+, F4/80+, and MHC-II+) polarization (24, 25). Cells were fixed with 1% paraformaldehyde and analyzed on a Gallios flow cytometer (Beckman Coulter, Brea, CA, USA).

## Results

### Environmental Scanning Electron Microscopy (E-SEM)

**Figure 1:**
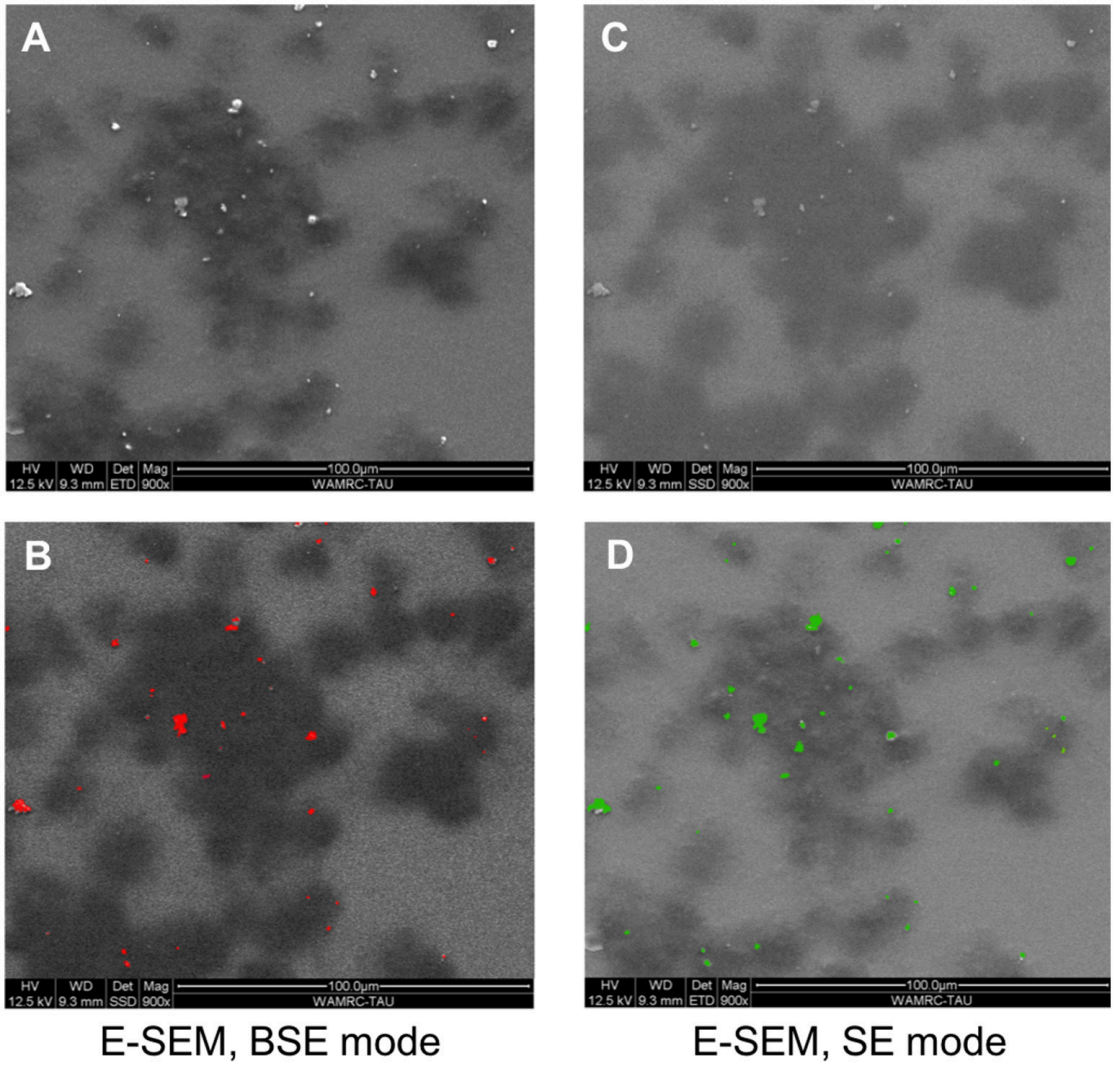
BMDM in the presence of Ti particles, visualized using E-SEM. BMDM were cultured for 24 h in the presence of SLA-derived Ti particles. Each field was taken either in backscattered electrons (BSE) mode or secondary electrons (SE) mode. Dark patches represent macrophages; light-bright dots **(A,C)** are Ti particles. **(B,D)** Color representation of the Ti particles (red in BSE mode, green in SE mode). Note that most particles are outside the cells, as indicated by the similarity between the BSE and SE modes.

To determine whether particles are internalized by the cells, a culture of BMDM and Ti particles was examined by E-SEM (Figure 1). Each field was taken either in BSE or SE mode. BSE provides a topographic view of the upper-most layer, which means that the Ti particles seen in this mode were not internalized by the cells but rather, were laid on top of the cell membrane (Figures 1A,B). The SE mode is a deep penetrating mode, able to detect all metals in the sample, within or outside the cells (Figures 1C,D). Based on the similarity between the two scanning modes (the total number of Ti particles and particles in the upper-most layer), it can be concluded that BMDM failed to internalize most of the US-released Ti particles.

### Effect of Ti Particles on Macrophage Polarization

**Figure 2:**
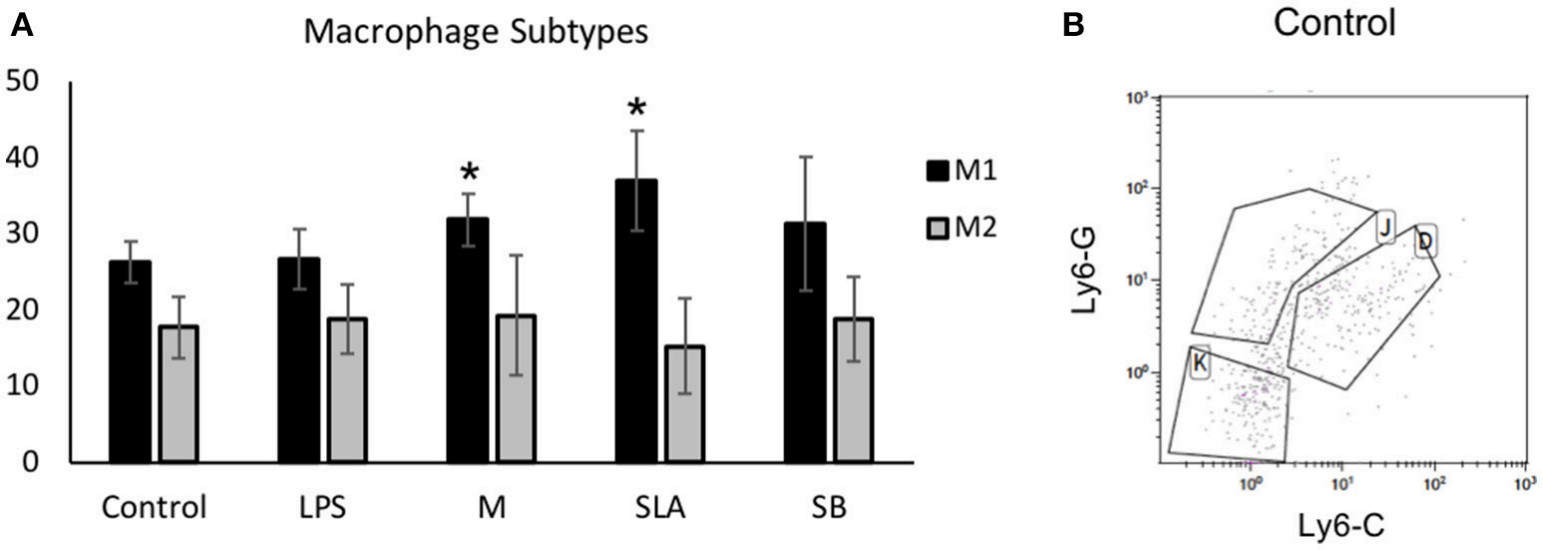
Ti particles induce macrophage polarization *in vivo*: Control fibrin membranes or membranes with either LPS or Ti particles were implanted onto mice calvaria (*n* = 5). Ti particles were obtained from machine (M), SLA or sand-blasted (SB) discs. Mice were euthanized after 3 weeks for soft tissue FACS analysis of macrophage polarization, using specific markers. Results show **(A)** the percentage of gated cells in the macrophages' sub-types, presented as mean ± SD; *p < 0.05 vs. control, 1-way ANOVA. **(B)** Representative FACS plot (K: monocytes, J: M2 and D: M1 macrophages).

The effect of Ti particles on macrophage polarization was examined *in vivo*, using a mouse calvarial model (12). Either Ti particles (from M/SLA/SB-treated discs) or LPS was incorporated into a fibrinogen-thrombin degradable membrane. Mice were euthanized 3 weeks post-op to examine the macrophages inside the soft tissue covering the parietal bones. Our FACS analysis first gated a CD11b+/F480+/MHC-II+ monocyte/macrophage population, which was further divided into Ly6G-/Ly6C-(Gr1-) non-inflammatory monocytes/macrophages (26), Ly6G+/Ly6C+ M1 and Ly6G+/Ly6C- M2 macrophages (24, 25). Our results show no appreciable difference in the M2 population in either group (Figure 2). However, the M1 population was significantly higher in animals exposed to either machined or SLA surface-derived Ti particles. Surprisingly, milder differences were recorded for the SB particles. LPS alone also did not cause an appreciable increase in the M1 macrophage population (Figure 2). It is reasonable to assume that the fibrinogen membrane by itself caused some inflammatory changes that masked the differences between LPS and the empty membrane (control).

### Cytokine Secretion Profile of Macrophages Exposed to Ti Particles

**Figure 3:**
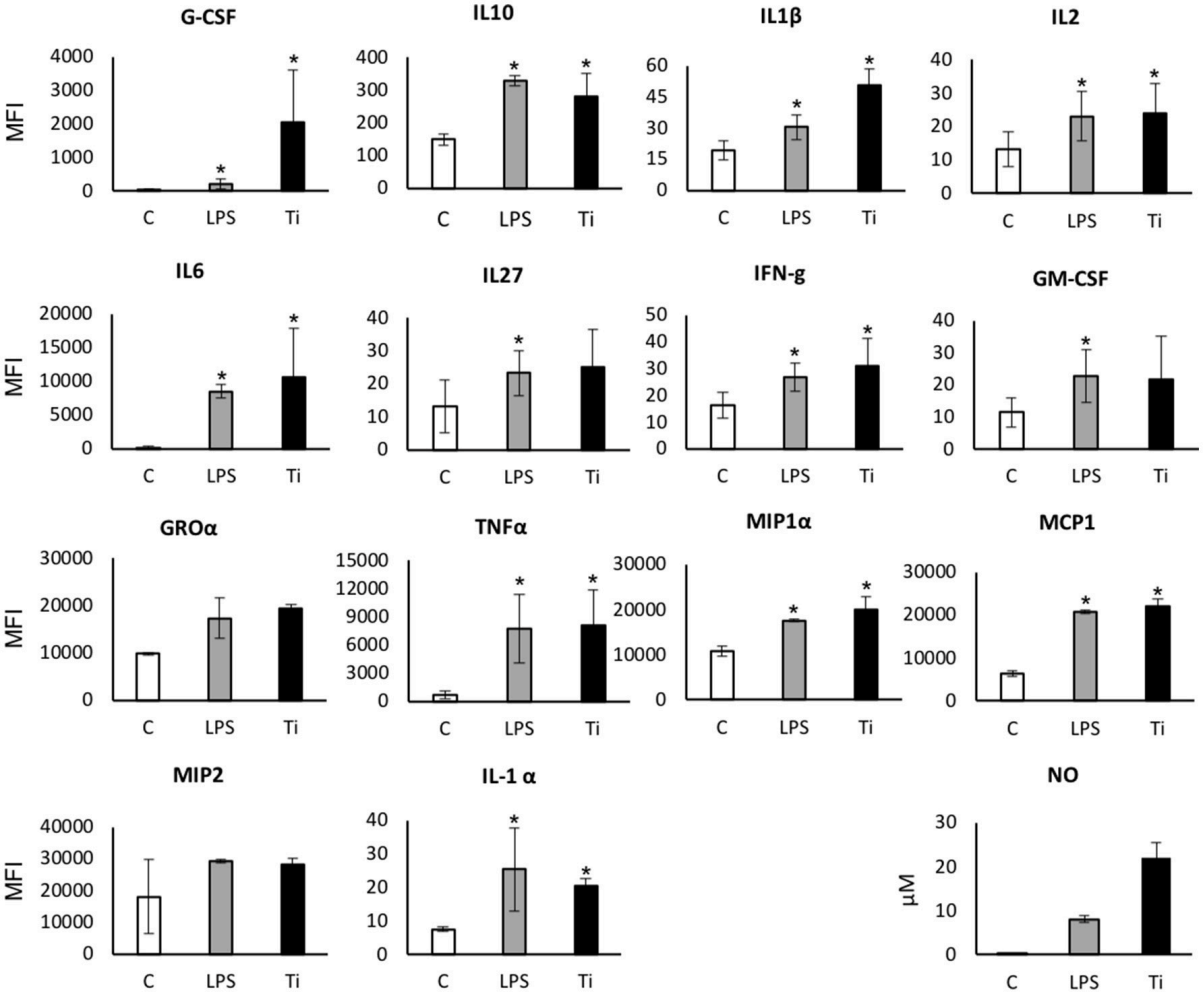
Secreted protein and NO levels in the supernatant of macrophages exposed to LPS/Ti particles. BMDM were cultured in the presence of either LPS or SLA-derived Ti particles. After 24 h, the supernatant was collected and analyzed using multiplex cytokine assay for secreted protein levels, and Griess Reagent System kit for NO. Results are presented as mean Melt Flow Index (MFI) units ±SD, and μM (for NO). *n* = 4. Experiment was repeated once with similar results. *p < 0.05 vs. control; non-parametric ANOVA.

To further reveal the mechanism underlying the particle-induced inflammatory response, the proteomic profile of macrophages exposed to Ti particles was assessed using the multiplex cytokine kit, after 24 h exposure to either LPS or SLA-derived particles. For most cytokines, the presence of Ti particles induced a 2- to 10-fold elevation in the proinflammatory cytokine levels (Figure 3). A modest (~2-fold) elevation in the anti-inflammatory IL1α and IL10 levels was also found, as well as in IFNγ. With respect to IL1β, IL6, and TNFα, our multiplex analysis showed a marked increase in their levels in the supernatant of macrophages cultured with Ti particles, in line with our previously published RT-qPCR analysis (12). Overall, this cytokine profile is characteristic of an M1 polarization, supporting our findings *in vivo* (Figure 2). We also observed a significant increase in NO secretion, which is a hallmark of M1 macrophage polarization (27).

### Anti-inflammatory and Anti-osteoclastogenic Effects of Neutralizing Antibodies in BMDM and Preosteoclast Cultures

**Figure 4:**
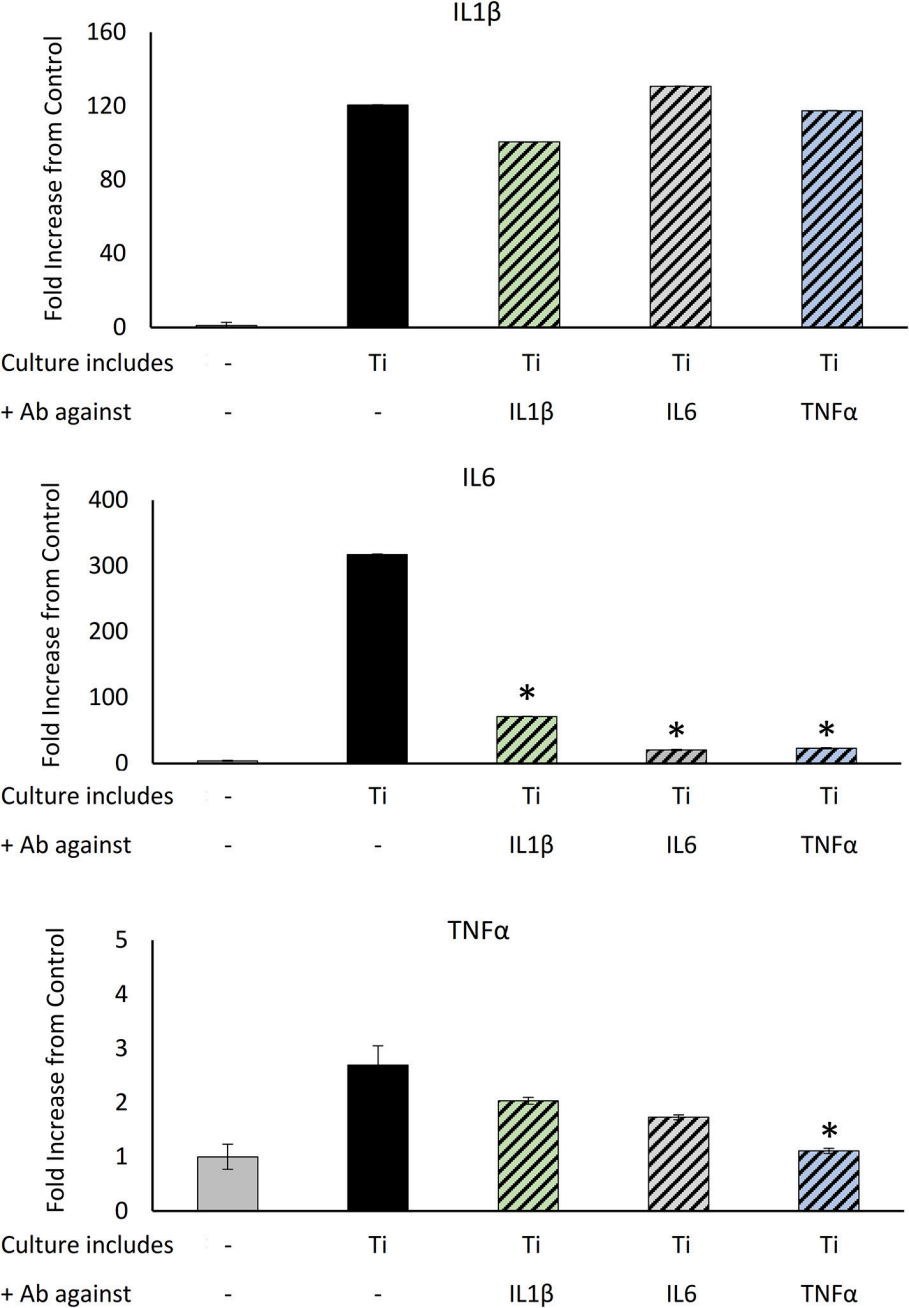
The effect of neutralizing Ab on Ti particle-induced inflammatory response: BMDM were cultured for 24 h in the presence of SLA-derived Ti particles released by US with neutralizing Ab for IL1β, IL6, or TNFα. IL1β, IL6, and TNFα expression levels were measured using RT-qPCR, normalized to GAPDH, and expressed as a fold change relative to the control (no particles). Data are shown as the mean ± SD. *p < 0.05 vs. Ti, non-parametric ANOVA, *n* = 3.

The concomitant increase in all inflammatory cytokines (Figure 3) raised a question regarding a positive paracrine feedback loop that fuels the inflammatory response of the macrophages. To determine whether blocking the paracrine effect of the cytokines secreted in response to Ti particles attenuates the overall inflammatory response of the macrophages, BMDM were cultured in the presence of Ti particles and treated with neutralizing antibodies for IL1β, IL6, or TNFα. Cells were collected after 24 h and changes in the levels of pro-inflammatory cytokines were measured using RT-qPCR. The secretion of IL1β was not affected by either of the neutralizing antibodies in the culture (Figure 4). On the other hand, IL6 expression in macrophages reduced significantly in the presence of each of the neutralizing antibodies. In contrast, TNFα expression in the culture was only affected by anti-TNFα antibodies. These findings suggest that the overall inflammatory response to Ti particles not only results from direct contacts between macrophages and Ti particles, but also results from positive feedback induced by paracrine signals.

**Figure 5:**
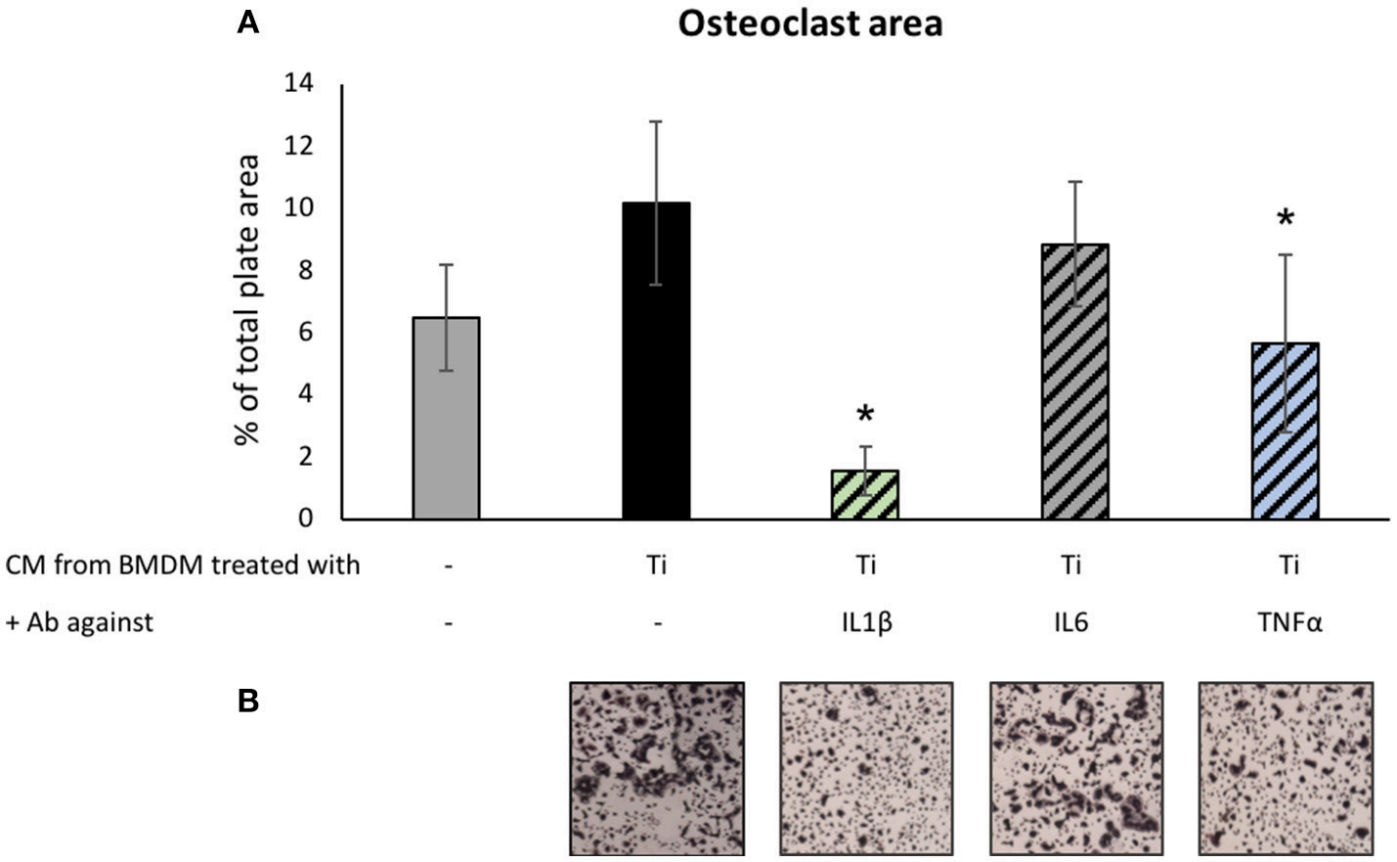
The effect of neutralizing Ab on Ti-particle-induced osteoclastogenesis: Preosteoclast cultures under osteoclastogenic conditions for 48 h were supplemented with condition medium (CM, supernatant) of BMDM cultured with or without SLA-derived Ti particles. At the same time, neutralizing antibodies against IL1β, IL6, or TNFα were added as indicated. Cultures were stopped after 36 h. **(A)** Average osteoclast area expressed as mean ± SD, *n* = 8. *p < 0.05 vs. Ti, 1-way ANOVA. **(B)** Representative images of TRAP-stained osteoclasts for each condition. Original magnification × 4.

Next, we tested the potential antagonizing effect of the same neutralizing antibodies on the osteoclastogenic signals emitted by macrophages in the presence of Ti particles. To this end, preosteoclasts cultured under osteoclastogenic conditions for 48 h were supplemented with the CM of BMDM cultured with or without Ti particles. Concomitantly, neutralizing antibodies against IL1β, IL6, or TNFα were added as indicated (Figure 5). Cultures were stopped on the 4th day, and the total area covered by multinucleated (>3 nuclei) TRAP-positive osteoclasts was calculated. Treatment with anti-IL6 antibodies did not affect osteoclastogenesis in our culture of isolated preosteoclasts. However, neutralizing antibodies against either IL1β or TNFα suppressed osteoclastogenesis in the presence of CM from BMDM exposed to Ti particles (Figure 5).

### Dynamics of Ti Particle-Induced Osteolysis

**Figure 6:**
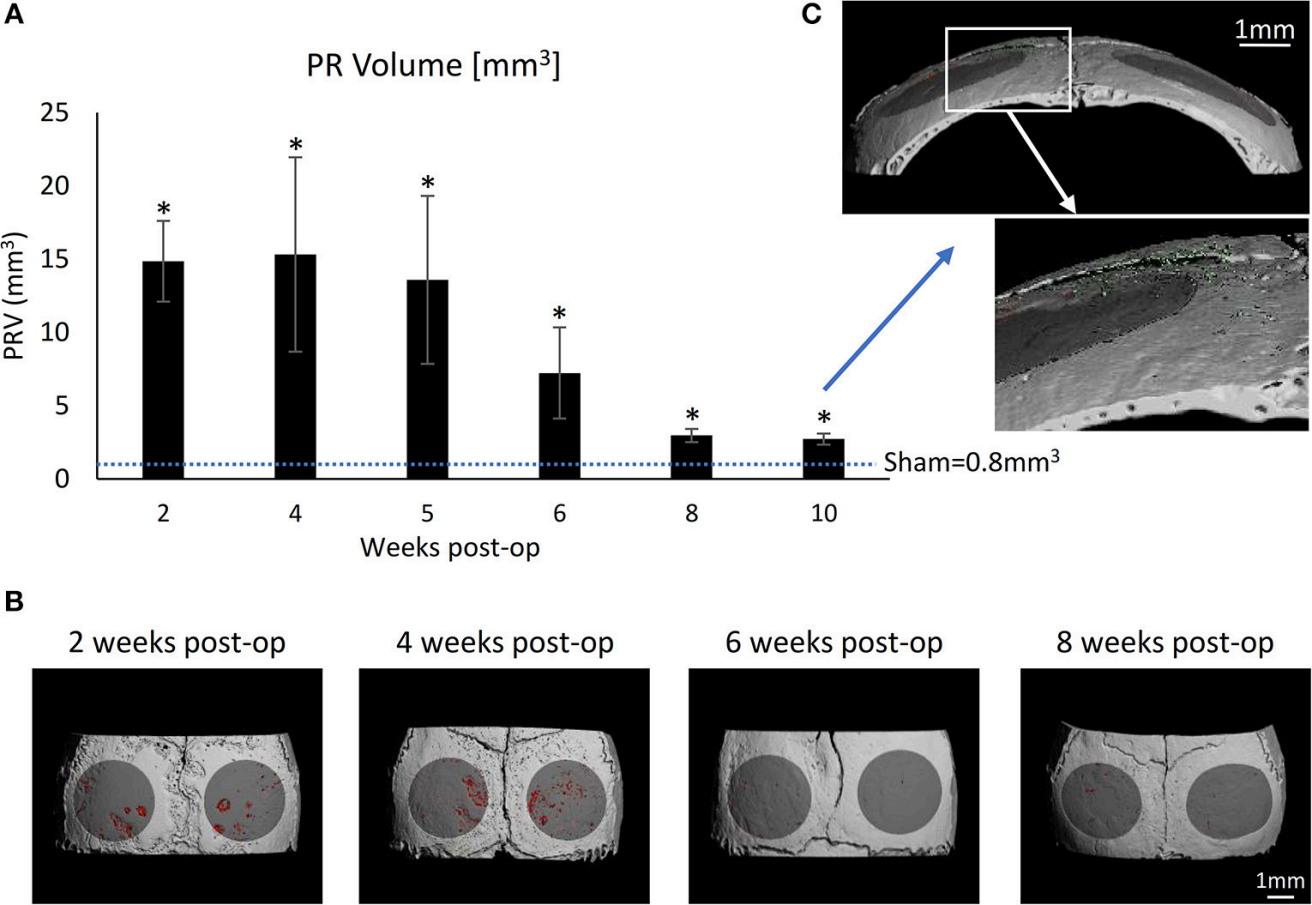
Time-lapse development of particle-induced bone resorption *in vivo*: Fibrin membranes loaded with SLA-derived Ti particles, were implanted onto mouse calvaria. Mice were euthanized every 2 weeks for μCT analysis to evaluate the dynamic progression of particle-induced osteolysis. **(A)** Pit Resorption Volume (PRV, mm^3^); data are expressed as mean ± SD, *n* = 5, *p < 0.05 vs. sham (no membrane), non-parametric ANOVA. **(B)** Representative μCT images of the calvaria are shown. The region of interest (ROI) is denoted in dark gray, and the resorption pits are denoted in red. **(C)** High magnification of a sample from the 10-week group indicating the residual Ti particles (green) despite the complete resorption of the membrane.

To account for the dynamics of the inflammatory response, we first established the time course of particle-induced osteolysis in our calvarial model. Ti particles released by the US scaling of SLA-treated discs were incorporated into the inserted membranes. A sham group consisted of mice undergoing the same surgical procedure without membrane insertion, which were euthanized after 4 weeks. Mice carrying a Ti particle-loaded membrane were euthanized every 2 weeks to monitor the extent of calvarial resorption using μCT. The results indicate that a dramatic resorption occurred during the first 5 weeks, along with a recovery from the 6th week onward. Of note, the membrane was totally degraded within 4 to 5 weeks; however, the presence of Ti particles was still detected even after 10 weeks (Figure 6C). This suggests that the osteolytic response is self-contained and somewhat recovers within 8 weeks after the last insult of Ti particles. Importantly, despite this recovery, the PR volume after 8 and 10 weeks remained significantly higher in the Ti than in the sham group.

Based on these results, further treatments were given for 4 weeks. After 4 weeks, the calvariae were collected and processed for μCT.

### Therapeutic Potential of Neutralizing Antibodies on Ti Particle-Induced Osteolysis

**Figure 7:**
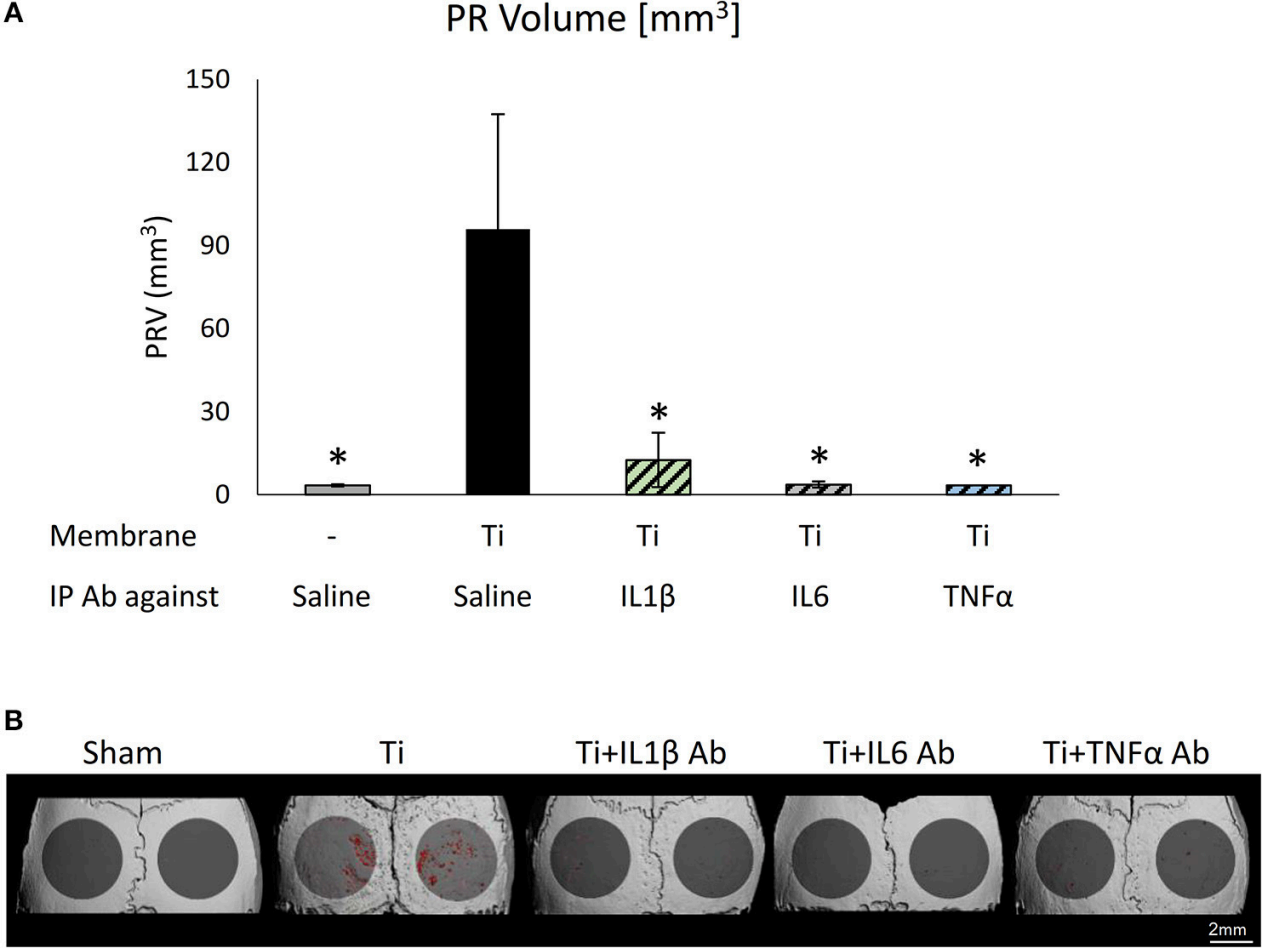
The effect of systemically administering anti-inflammatory neutralizing antibodies on particle-induced osteolysis *in vivo*: Fibrin membranes including SLA-derived Ti particles were implanted onto mouse calvaria. Neutralizing antibodies (Ab) against IL1β, IL6, or TNFα (or saline as control) were injected intraperitoneally (IP) 1 day before, once a week (IL6 and TNFα), or daily (IL1β) after membrane insertion. Mice were euthanized 4 weeks post-op for μCT analysis. **(A)** Pit Resorption Volume (PRV, mm^3^), data are expressed as mean ± SD, *n* = 6; *p < 0.05 vs. Ti, 1-way ANOVA. **(B)** Representative μCT images of the calvaria, color-coded as above.

We repeated the same calvaria model with membranes loaded with SLA-derived Ti-particles. Neutralizing antibodies for IL1β, IL6, or TNFα (or saline as control) were injected intraperitoneally 1 day before, once a week (IL6 and TNFα), or daily (IL1β) after the membrane insertion. As shown in Figure 7, each of the 3 neutralizing antibodies had a dramatic, statistically significant effect in preventing particle-induced osteolysis.

**Figure 8:**
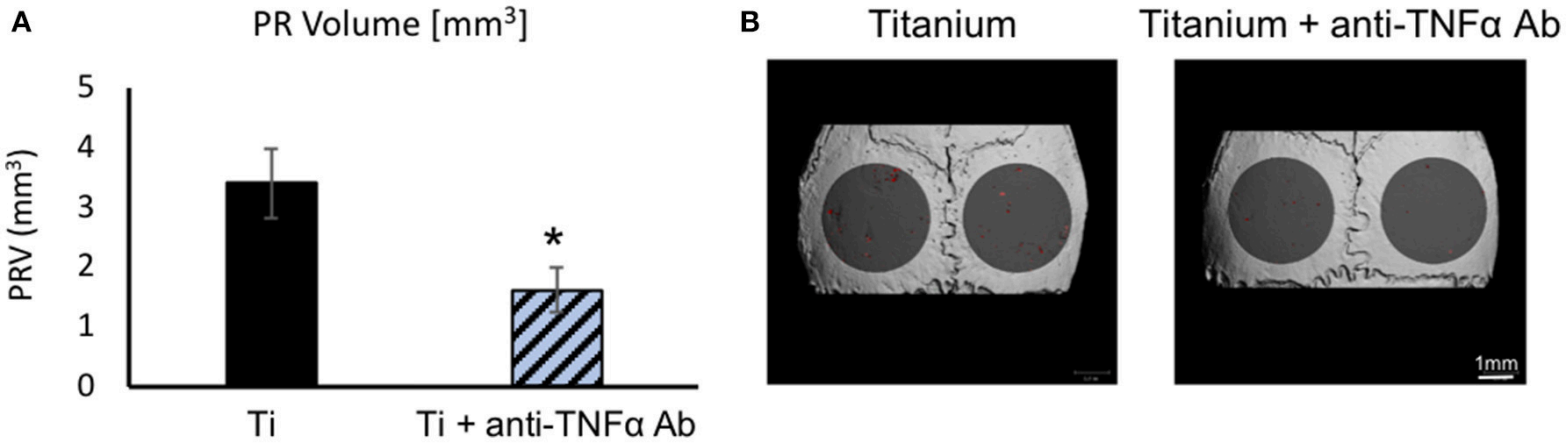
The effect of topically-administered anti-inflammatory neutralizing antibodies on particle-induced osteolysis *in vivo*: Fibrin membranes including Ti particles, together with neutralizing antibodies against TNFα (or saline as control), were implanted onto mouse calvaria. Mice were euthanized 4 weeks post-op for μCT analysis. **(A)** Pit Resorption Volume (PRV, mm^3^), data are expressed as mean ± SD, *n* = 6; *p = 0.029 vs. Ti, *t*-test. **(B)** Representative μCT images of the calvaria color-coded as above.

Next, we tested the therapeutic potential of topically administered neutralizing antibodies. As a proof-of-concept, we elected to test anti-TNFα neutralizing antibodies incorporated into the membrane together with the Ti particles (Figure 8). Importantly, we found that topically-administered neutralizing antibodies significantly suppressed the particle-induced osteolysis (Figure 8).

## Discussion

In this study, we examined the mechanism by which Ti particles lead to inflammation and bone resorption and tested the therapeutic potential of using specific neutralizing antibodies in preventing particle-induced osteolysis.

Our multiplex analysis revealed an interesting secretion profile of macrophages cultured in the presence of Ti particles. There was a dramatic elevation in the levels of most pro-inflammatory cytokines relative to the modest rise in the anti-inflammatory IL1α and IL10 levels, which may be a compensatory restraining response (28, 29). IFNγ also displayed a significant rise; however, its contribution to the inflammatory response remains unclear, since both pro- and anti-inflammatory actions have been reported for this cytokine (30–32). MIP1α and MCP1 are chemokine proteins (CCL3 and CCL2, respectively). MIP1α is involved in the acute inflammatory state and is responsible for recruitment of polymorphonuclear cells, whereas MCP1 was found in the vicinity of bone resorption sites (33). The latter chemokine may drive osteoclast differentiation in the absence of RANKL (34). With respect to IL1β, IL6, and TNFα, our multiplex analysis revealed a trend very similar to our RT-qPCR analysis reported recently (12), with significantly elevated levels in the supernatant of macrophages cultured with Ti particles. In general, Ti particles induce in macrophages a response similar to that of LPS. The resulting inflammatory response drives the bone tissue damage mediated by osteoclasts. Together with the FACS, gene expression and secretome profiling on macrophages *in vitro* and *in vivo*, these changes indicate that macrophages undergo an “M1-like” polarization in response to Ti particles. It should be noted however that all our assays were conducted at the early stages of the inflammatory response. The resolving inflammation manifested in the partial tissue repair observed *in vivo* after 6–8 weeks (Figure 6), suggests a more dynamic and complex spatio-temporal distribution of M1 and M2 macrophages (27).

**Figure 9:**
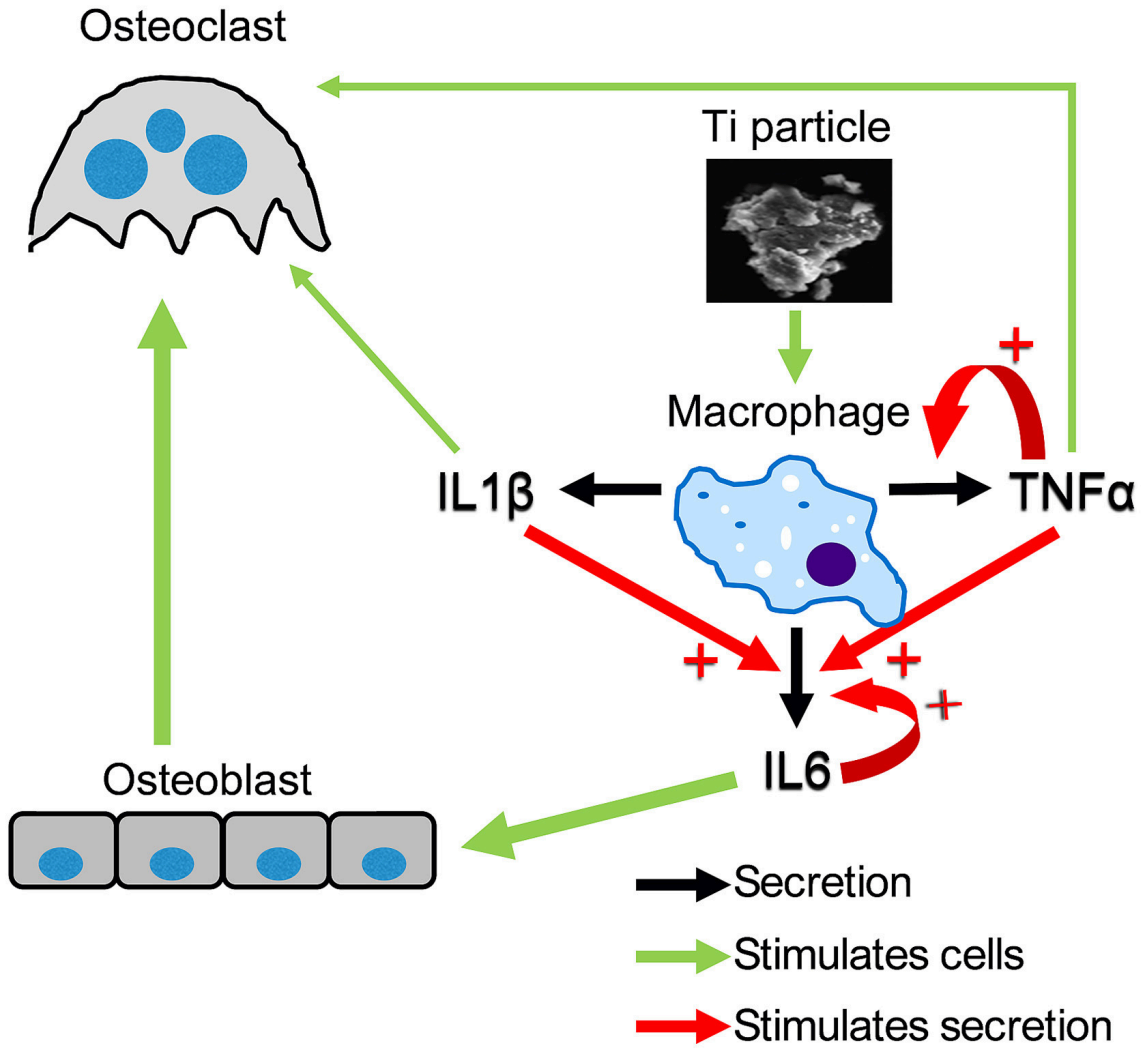
Putative model of the hierarchical roles of IL1β, IL6, and TNFα in particle-induced osteoclastogenesis. This model describes the paracrine and autocrine induction of cytokine secretion by macrophages exposed to SLA-derived Ti particles. The thickness of the green arrows reflects the importance of one pathway over the others.

Next, we examined how neutralizing antibodies against IL1β, IL6, and TNFα affected Ti particle-induced inflammatory response in macrophages. Importantly, we found that (i) the secretion of IL1β was not affected by either of the neutralizing antibodies, (ii) IL6 expression was significantly decreased by the presence of each of the neutralizing antibodies, and (iii) TNFα expression in culture was only affected by anti-TNFα antibodies. This is in accordance with the notion that both TNFα and IL1β are upstream factors in the inflammatory cascade (35). Indeed, our findings suggest that IL1β expression is independent of the secretion of IL1β, IL6, and TNFα by neighboring macrophages. In contrast, most IL6 expression by macrophages depends on paracrine signals, including IL1β, IL6, and TNFα. TNFα expression is only partly dependent on these signals, since only blockade of TNFα partly suppressed its own expression. Importantly, we also observed that *in vivo*, blocking any of these 3 cytokines significantly attenuated bone resorption. Based on these observations we propose a model to describe this additive and synergistic interrelationship between these 3 cytokines and the resulting stimulation of osteoclasts, thus inducing bone resorption (Figure 9). In this model, blocking IL1β does not affect TNFα expression and vice versa, and blocking IL6 does not affect either IL1β or TNFα expression. Moreover, both IL1β and TNFα directly stimulate osteoclasts while IL6 does so indirectly via the osteoblasts. The latter is suggested by the significant blockade of osteolysis by anti-IL6 antibodies administered *in vivo*, but not *in vitro* in the absence of stromal cells in the cultures. This conclusion is also in line with another study demonstrating that IL6 stimulates osteoclastogenesis via osteoblasts (36). Overall, our model suggests that Ti particles stimulate osteoclastogenesis via 3 pathways, the predominant one being the synergistically increased expression of IL6 by IL1β and TNFα, which in turn stimulates osteoblast-mediated osteoclastogenesis. It is reasonable to assume that both *in vitro* and *in vivo*, not all macrophages are in direct contact with Ti particles. The paracrine effect, depicted here, portrays a chain reaction that could provide the inflammatory signals with an extended range.

We conducted here a time-lapse experiment and evaluated the long-term effects of Ti particles on bone resorption *in vivo* (Figure 6). Our data show that the osteolytic response is self-contained and somewhat recovers, although not entirely. After 8 and 10 weeks, the extent of bone residual defects remained significantly higher than in the sham group. Moreover, in clinical settings, a Ti prosthesis is likely to continuously release ions, debris, and particles, thus fueling the inflammatory response, and further aggravating osteolysis.

Previous articles studied different approaches to block the progression of bone resorption. These approaches included promoting apoptosis of osteoclasts, genetic intervention (37, 38) or using bisphosphonates, which caused pathological fractures (39).

In our previous publication, we characterized the size, number, physical and chemical properties of the titanium particles shedding from the surface of rough titanium implant commonly used in contemporary dentistry following ultrasonic scaling (12). Regarding the size, we used an automated cell counter to measure the number and size of the particles shedding from one 6-mm diameter titanium disc with an SLA surface. We found that ultrasonic scaling of each SLA disc generates 2.54 million titanium particles of an average size of 6 to 12 μm. A previous study established that the range of wear debris generated from orthopedic prostheses is between 1 and 30 μm (40). It is thus likely to assume that the cellular and inflammatory responses described in the current study with ultrasonic-generated particles are similar to those observed with wear debris surrounding orthopedic prostheses.

Three main biological strategies were tested to block the chronic inflammatory reaction to orthopedic wear particles, including (i) interference with systemic macrophage trafficking to the local implant site, (ii) modulation of macrophages from an M1 to an M2 phenotype in periprosthetic tissues, and (iii) local inhibition of the transcription factor nuclear factor-kappa B, thereby interfering with the production of pro-inflammatory mediators (38). All three approaches showed promising results in preclinical studies but have not yet been evaluated clinically.

Here we tested three clinically-approved neutralizing antibodies that are prescribed for the management of auto-immune and inflammatory diseases. Anti-IL1β antibodies (Anakinra) are prescribed to rheumatoid arthritis and neonatal-onset multisystem inflammatory disease, anti-IL6 receptor antibodies (Tocilizumab) are administered to treat arthritis and anti-TNFα antibodies (Adalimumab) are effective against rheumatoid arthritis, psoriasis and Crohn's disease (41–43). Our *in vivo* and *in vitro* results showed a clear association between each of these cytokines and the macrophage response to Ti particles. Using our mouse calvarial model, we demonstrated that blocking each of these cytokines prevents Ti particle-induced osteolysis. We further showed that both systemic and local administration are conceptually possible approaches. This preclinical study therefore advocates that neutralizing antibodies be further tested against IL1β, IL6, or TNFα in clinical settings for managing the aseptic loosening of orthopedic prostheses and oral peri-implantitis.

## Author Contributions

ME, SH-B, TL, NS, YC, DK, and YG: contributed to the design of the research; ME, SH-B, and TL: performed research and contributed to the obtained results; YG and DK: supervised the project; ME and YG: wrote the paper and prepared it for publication. All authors read and approved the final manuscript.

### Conflict of Interest Statement

The authors declare that the research was conducted in the absence of any commercial or financial relationships that could be construed as a potential conflict of interest.
